# Galectin-3 is Associated with Heart Failure Incidence: A Meta-Analysis

**DOI:** 10.2174/1573403X19666221117122012

**Published:** 2023-03-22

**Authors:** Basil M. Baccouche, Mattia A. Mahmoud, Corrine Nief, Karan Patel, Barbara Natterson-Horowitz

**Affiliations:** 1 Stanford University School of Medicine, Stanford, California, USA;; 2 Department of Public Health and Primary Care, University of Cambridge, Cambridge, United Kingdom;; 3 Perelman School of Medicine, Philadelphia, Pennsylvania, USA;; 4 Cooper Medical School, New Jersey, USA;; 5 Department of Medicine, Harvard Medical School, Boston, Massachusetts, USA

**Keywords:** Incident HF, heart failure, cardiovascular disease, biomarker, galectin-3, hazard ratio, risk ratio

## Abstract

**Introduction:**

Heart failure (HF) is a leading cause of death worldwide. The global prevalence of heart failure is projected to increase rapidly in the coming decades, and significant attention has turned to improving biomarker-based risk prediction of incident HF. This paper aimed to qualitatively and quantitatively evaluate the evidence associating levels of galectin-3 with the risk of incident HF.

**Methods:**

A review of PUBMED-indexed peer-reviewed literature was performed. Nine studies met the inclusion criteria, and all nine had data eligible for conversion and pooling. A random-effects meta-analysis was performed using hazard ratios and 95% confidence intervals from a minimally adjusted model, a further adjusted model, and from subgroups within the further-adjusted model.

**Results:**

The minimally-adjusted model provided an HR of 1.97 (95% CI 1.74-2.23) when comparing the top quartile of log-gal-3 to the bottom quartile. The further-adjusted model provided an HR of 1.32 (95% CI 1.21-1.44) for the same comparison. The positive, significant association was conserved during sensitivity analysis.

**Conclusion:**

There is a significant positive association between circulating galectin-3 and the risk of incident heart failure. Given the complex mechanistic relationship between galectin-3 and cardiovascular pathophysiology, further investigation is recommended for the possible implementation of galectin-3 into clinical risk prediction models.

## 1 INTRODUCTION

### 1.1 The Burden of Heart Failure

Cardiovascular disease (CVD) is the most common cause of death in the United States [1, 2]. Heart failure (HF) is a chronic progressive form of CVD wherein ventricular filling or ejection of blood is impaired [3-6]. Heart failure is a pandemic affecting tens of millions, with high morbidity and mortality [7]. The global prevalence of heart failure is projected to increase rapidly in the coming decades [7].

### 1.2 Galectin-3: Protein and Biomarker

Galectin-3, a protein of the galectin family causally responsible for several physiological (and pathophysiological) processes within the cardiovascular system relating to fibrosis, atherosclerosis, and heart failure, has emerged as a potential biomarker for the incidence of certain cardiovascular diseases [8, 9]. In particular, galectin-3 is characteristically overexpressed by “profibrotic” M2 macrophages and is implicated in the tissue fibrosis response *via* sustained myofibroblast and macrophage activation by intracellular and extracellular signaling pathways [9]. Normal cardiac tissue expresses very little galectin-3, but cardiac injury results in rapid galectin-3 upregulation [9]. Galectin-3 injection in adult male rats has been demonstrated to produce cardiac dysfunction and remodelling of ventricular tissue, and genetic deletion of galectin-3 has been shown to reduce cardiac fibrosis and inflammation in mice [9]. In humans, circulating levels of galectin-3 can be measured from serum or plasma samples [9].

As many developing nations experience a shift towards a higher burden of non-communicable, chronic diseases, the importance of improving biomarker-based heart failure risk prediction continues to grow [10]. Although successful targeted pharmacological therapeutics have been developed for certain subtypes of HF, few effective therapies exist for other subtypes, notable among them HFpEF [11]. A potential limiting factor for developing preventative and acute therapies may be an incomplete biomarker-based understanding of this relationship.

This article aims to rigorously evaluate and quantify the published evidence associating galectin-3 levels with the incidence of heart failure. This is done to meet two primary objectives. The first objective is to reduce uncertainty surrounding the role of galectin-3 in incident heart failure *via* a meta-analysis of the growing amount of evidence associating galectin-3 and incident heart failure. As a result of establishing an association between galectin-3 levels and incident heart failure, the second objective is to support a future investigation of galectin-3 as a possible predictive biomarker of incident heart failure, a clinically useful possibility that has thus far been met with a limited inquiry. Studies have been performed with mixed results assessing the value of galectin-3 in predicting mortality in patients with HF [8]. To date, the authors have identified no review and meta-analysis of the literature associating galectin-3 levels and incident HF in the peer-reviewed literature.

## 2 METHODS

### 2.1 Search Strategy

A review examining the evidence associating galectin-3 and the incidence of heart failure was performed using the Sciome Workbench for Interactive computer-Facilitated Text-mining (SWIFT)-Review, which uses statistical text mining to sort search results for high-efficiency manual screening [12, 13]. The following search terms were processed using the United States National Library of Medicine’s PubMed database: (HF Incidence OR heart failure incidence) AND (galectin-3 OR gal-3). No restrictions were applied to the search.

**Fig. (1):**
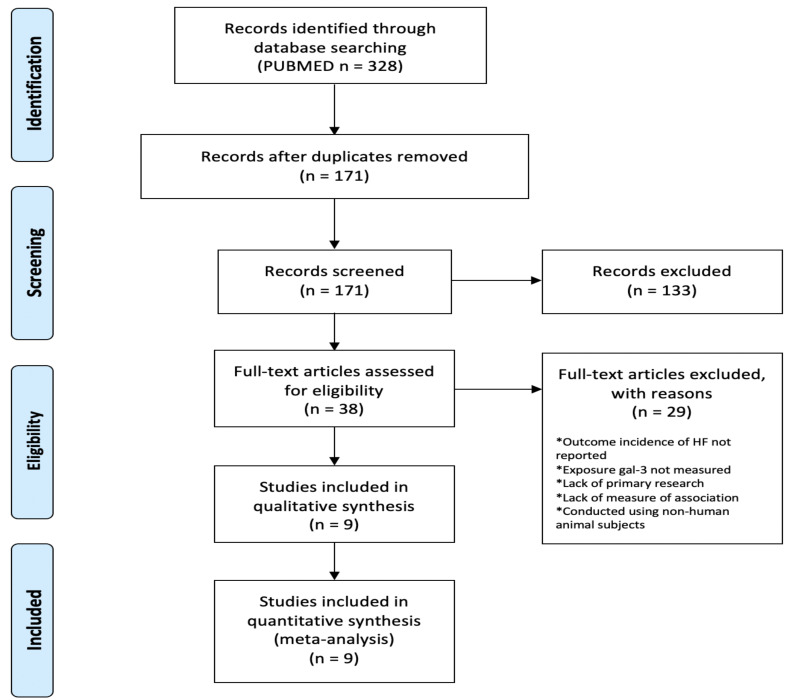
Reproducible, PRISMA-compatible review workflow [14].

The 171 results were manually screened by title and abstract using our predefined inclusion criteria (Table **1**) and in accordance with the reproducible PRISMA-compatible flow process (Fig. **1**) [14].

If multiple papers used the same population cohort, the study with the most relevant data was included. The study by de Boer *et al*. in 2018 (Table **2**) includes minor overlap from community-based populations used for other studies (FHS and PREVEND) [15]. However, descriptive data for novel study populations CHS and MESA were less comprehensive and, therefore, less suitable for meta-analysis than the pooled overall data. Supplementary materials were consulted when performing a full-text evaluation.

### 2.2 Data Collection and Quality Assessment

Study design characteristics and relevant data were manually extracted from full-text reviews (including supplementary materials) of included studies. Galectin-3 data were collected from reported serum or plasma galectin-3 assays. Incidence of heart failure was considered the outcome of interest, defined by the included studies using respective study criteria or by hospitalization due to HF. Samples were drawn from general populations and populations with pre-existing kidney disease, diabetes, or myocardial infarction. Hazard and odds ratios were considered valid measures of association between galectin-3 and HF incidence. Odd and hazard ratios can be used interchangeably for rare diseases.

The Newcastle-Ottawa Scale was used to evaluate the quality of included non-randomized studies (Appendix I). Low, moderate, and high-quality studies were given scores between 0-3, 4-6, and 7-9, respectively. The complete results of this evaluation are shown in Appendix II.

### 2.3 Statistical Analysis

The study results were analyzed using a random effects meta-analysis due to inter-study heterogeneity. 1 standard-deviation log-gal-3-based hazard ratios and tertile-based hazard ratios were converted to quartile-based hazard ratios assuming that the log-risk ratio is linear and that log-gal-3 is normally distributed [16].

Heterogeneous hazard ratios across studies were converted and standardized for meta-analysis using the *riskconv* command in STATA 16.1 [17]. All meta-analyses were performed using STATA 16.1. Two-sided p-values below 0.05 were considered significant unless otherwise indicated. A risk ratio whose lower 95% confidence interval remains above a value of 1 is also considered significant.

Low numbers of studies with appropriate subgroups prevented meaningful stratified meta-analysis by sex, pre-existing CVD, or location. Sensitivity analyses were performed by location, pre-existing cardiovascular disease, and to exclude outliers

## 3 RESULTS

### 3.1 Overview of Included Studies

Nine studies were included after the literature review, consisting of eight prospective cohort studies and 1 prospective nested case-control study. Sample sizes ranged from 924 to 22,756 participants, and the mean participant age ranged from 48.8 to 74.8 years. Most studies included people with diabetes in their patient population, but only one study [18] drew from a patient population with pre-existing CVD. Another study [19] drew from a patient population with chronic renal insufficiency. Studies were conducted in the United States and Europe. Study characteristics are displayed in Table **2**.

All studies measured incident HF as an outcome of interest, and the number of HF events ranging from 166 to 2095, as shown in Table **3**. In eight of the nine studies, heart failure was clearly defined using the Framingham criteria, ESC guidelines, MORGAM/AHA criteria, or ICD-9 codes (Table **3**). Only one study [19] used unspecified “standardized clinical criteria” to define HF. Seven studies measured galectin-3 using an enzyme-linked immunosorbent assay (ELISA) manufactured by either BG Medicine or R&D Systems, and two used a chemiluminescent immunoassay (CMIA) manufactured by Abbott Diagnostics (Table **4**).

The main findings were reported as hazard ratios in the eight prospective cohort studies and as an odds ratio in the prospective nested case-control study (Table **5**). Studies in which the hazard ratio was reported by comparing 1 standard deviation log-gal-3 increase, the 3rd tertile of gal-3 compared to bottom tertile, or linear gal-3 doubling to incident HF were converted to 4th quartile-to-bottom-quartile log-gal-3 to incident HF hazard ratios for the purpose of meta-analysis [20]. The converted values are displayed in Appendix III.

The Newcastle-Ottawa Scale was used to quantify the methodological quality of each of the studies included (Appendix I & II). All included studies received a score of 7 or higher, indicating high quality. The limitations of each study are listed in the Discussion (Table **6**).

#### 3.1.1 Meta-Analysis

After conversion, all nine included studies reported data conducive to further-adjusted meta-analysis, but only eight reported data conducive to minimally-adjusted meta-analysis. De Boer *et al*., 2018 [15] were excluded from the minimally adjusted meta-analysis because they did not publish a minimally adjusted model.

**Fig. (2):**
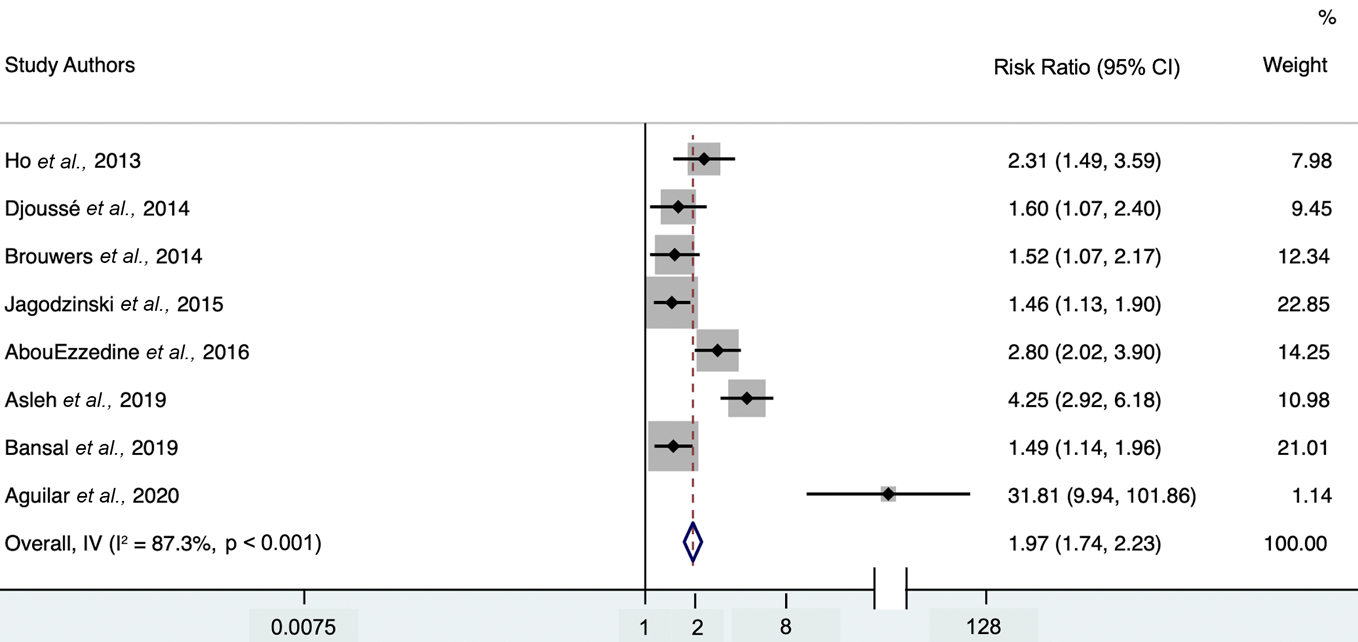
Forest plot of minimally adjusted model hazard ratios for the association between 4th-quartile-to-bottom-quartile log-gal-3 and incident HF. Study weights (represented by the grey boxes) are from the random-effects analysis. 95% confidence interval for the heterogeneity statistic I^2^: (76.7-91.9).

**Fig. (3):**
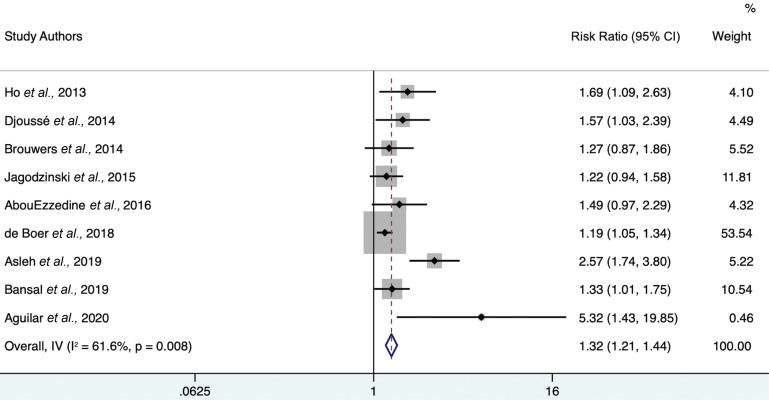
Forest plot of further adjusted model hazard ratios for the association between 4th-quartile-to-bottom-quartile log-gal-3 and incident HF. Study weights (represented by the grey boxes) are from the random-effects analysis. 95% confidence interval for the heterogeneity statistic I^2^: (0-79.7).

Each study individually demonstrated a positive association between 4th-quartile log-gal-3 levels and the risk of heart failure. The minimally adjusted random-effects model (Fig. **2**) provided a hazard ratio of 1.97 (95% CI 1.74-2.23) with heterogeneity statistic I^2^ = 87.3% (95% CI 76.7-91.9), indicating high heterogeneity. The maximally adjusted random-effects model (Fig. **3**) provided a hazard ratio of 1.32 (95% CI 1.21-1.44) with heterogeneity statistic I^2^ = 61.6% (95% CI 0-79.7), indicating moderate heterogeneity. Both the minimally and further-adjusted meta-analyses demonstrate a robust, significant positive association.

The study by Aguilar *et al*. in 2020 [26] was an obvious outlier. Due to its low weight, the overall further-adjusted hazard ratio (1.31 (95% CI 1.20-1.43)) did not change significantly upon its exclusion, as shown in Fig. (**4**). However, outlier exclusion did reduce the observed heterogeneity (I^2^ = 57.5% (95% CI 0-78.8)).

#### 3.1.2 Sensitivity Meta-Analysis

**Fig. (5):**
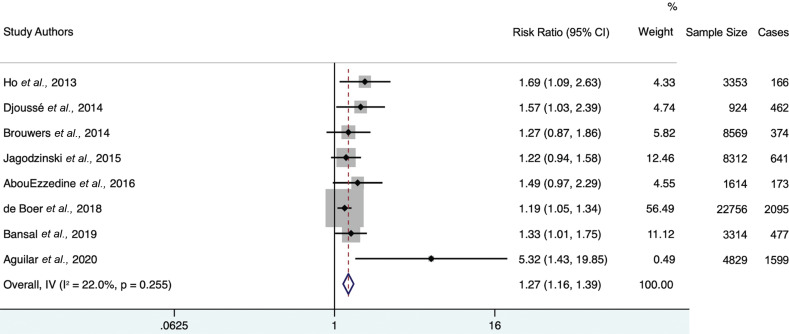
Forest plot of further adjusted model hazard ratios for the association between 4th-quartile-to-bottom-quartile log-gal-3 and incident HF, excluding study populations with pre-existing CVD. Study weights (represented by the grey boxes) are from the random-effects analysis. 95% confidence interval for the heterogeneity statistic I^2^: (0-65.3).

All sensitivity meta-analyses were performed using further-adjusted models. Only one study [18] utilized a study population with pre-existing CVD. Upon exclusion of this study (Fig. **5**), the overall hazard ratio was very modestly lowered from 1.32 (95% CI 1.21-1.44) to 1.27 (95% CI 1.16-1.39). Heterogeneity statistic I^2^ = 22% (95% CI 0-65.3), indicating low heterogeneity.

**Fig. (6):**
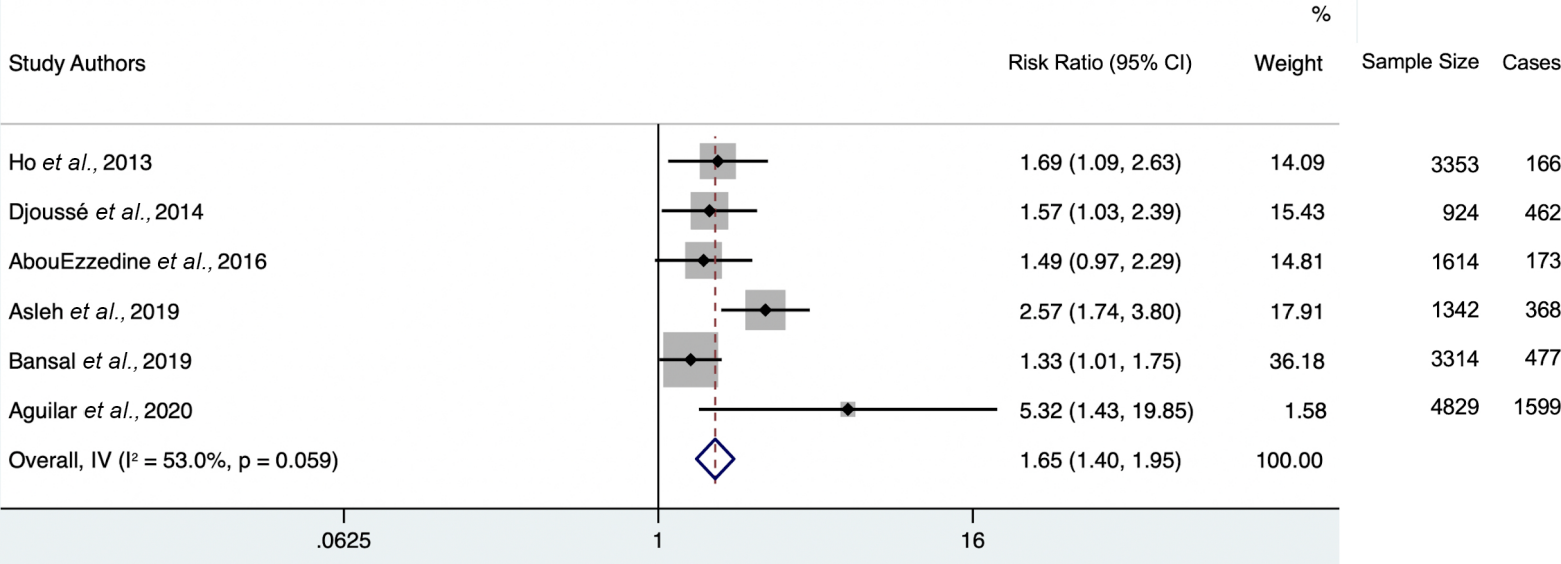
Forest plot of further adjusted model hazard ratios for the association between 4th-quartile-to-bottom-quartile log-gal-3 and incident HF, including only studies whose patient populations are exclusively within the United States. Study weights (represented by the grey boxes) are from the random-effects analysis. 95% confidence interval for the heterogeneity statistic I^2^: (0-79.3).

Six of the nine studies utilized study populations exclusively within the United States. Meta-analysis of USA-based studies (Fig. **6**) notably elevated the overall hazard ratio from 1.32 (95% CI 1.21-1.44) to 1.65 (95% CI 1.40-1.65). Heterogeneity statistic I^2^ = 53% (95% CI 0-79.3), indicating moderate heterogeneity.

**Fig. (7):**
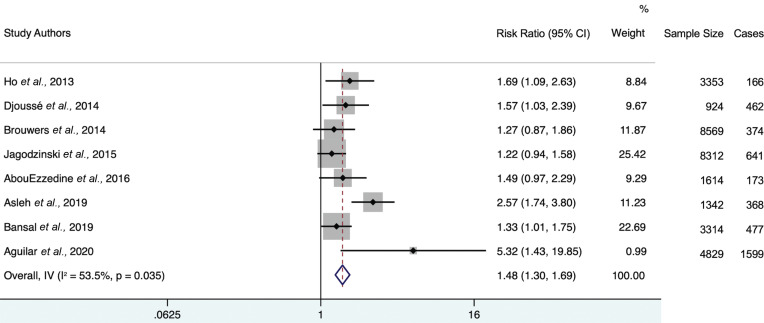
Forest plot of further adjusted model hazard ratios for the association between 4th-quartile-to-bottom-quartile log-gal-3 and incident HF, excluding the study with the highest weight (de Boer *et al.,* 2018). Study weights (represented by the grey boxes) are from the random-effects analysis. 95% confidence interval for the heterogeneity statistic I^2^: (0-77.2).

Of all included studies, de Boer *et al*., 2018 [15] had the largest weight percentage within the further-adjusted meta-analysis (53.54%). The exclusion of this study (Fig. **7**) elevated the overall hazard ratio from 1.32 (95% CI 1.21-1.44) to 1.48 (95% CI 1.30-1.69). Heterogeneity statistic I^2^ = 53.5% (95% CI 0-77.2), indicating moderate heterogeneity.

## 4 DISCUSSION

### 4.1 Summary of Findings

This review and meta-analysis demonstrate a significant association between the top- to bottom-quartile log-galectin-3 and the risk of heart failure. Adjusted meta-analysis presents a 32% higher HF risk in individuals with top-quartile log-galectin-3 levels than in the bottom quartile. Sensitivity analysis revealed that the significant positive association was conserved or even elevated upon outlier removal, location standardization, and removal of the study population with pre-existing CVD. Heterogeneity between studies was notable but decreased upon adjustment and further upon outlier and pre-existing CVD removal.

### 4.2 Study Limitations

The authors did not review preprint servers or non-PUBMED-indexed journals. As a result, it is possible that relevant data (even if not yet peer-reviewed) was overlooked.

The study by de Boer *et al*. in 2018 [15] accumulated data from four prospective cohort studies, two of which (FHS and PREVEND) had participants included in other studies. However, data on the individual unique cohorts (CHS and MESA) had significant descriptive omissions compared to information published on the pooled results, so pooled data were included despite the minor overlap.

Across the nine studies, different sets of criteria were applied to diagnose HF, introducing possible inter-study heterogeneity in HF diagnosis. Within the MESA cohort, which contributed to the pooled de Boer *et al*. [15] data, outcome ascertainment was dependent upon the conclusions of various medical records and not standardized to one uniform set of criteria, introducing possible inter-record heterogeneity in HF diagnosis.

The studies included in the minimally adjusted meta-analysis are heterogeneous in their choice of covariate adjustment; while a plurality adjusted for age/sex, others were unadjusted or made significant adjustments. The studies included in the further-adjusted meta-analyses are also heterogeneous in their choice of covariate adjustment.

Heterogeneity between studies, as defined by the I^2^ statistic, was moderate to high in most studies. Unfortunately, given the recency with which the association between galectin-3 and incident HF has been investigated, the number of studies to choose from is limited and heterogeneous.

The relative paucity of published research on the association in question meant that the number of meta-analyzed studies (9) was low. In addition, the scarcity of available research meant that our outcome of interest (incident HF) could not be further specified by subtype (HFrEF and HFpEF).

Although robust associations were observed between circulating galectin-3 levels and incident heart failure, statistical causality was not established by this study and is recommended as a promising future direction of research to resolve the ongoing debate surrounding the possible role of gal-3 in heart failure onset.

Finally, each included study's limitations are represented below in Table **6**.

### 4.3 Study Strengths

This review synthesizes and meta-analyzes data on the association between galectin-3 and incident HF. Interest in the relationship between galectin-3 exposure and incident HF outcome has only occurred within the last decade or so. As a result, this review provides an up-to-date synthesis of established knowledge. Where specified, Galectin-3 was measured ubiquitously within the same sample medium (plasma), and assay manufacturers were reputable. Individual study sample sizes are generally high. All included studies had data compatible with meta-analysis, and there were no exclusions in the further-adjusted model. In addition, further-adjusted models were rigorously subjected to sensitivity analysis by location, pre-existing cardiovascular disease, and outlier studies. The overall association was robust and highly significant. Finally, applying the Newcastle-Ottawa Scale to our studies (Appendix I & II) demonstrated that all nine included studies were considered “high” quality.

### 4.4 Implications

Heart failure is a complex physiological dysfunction with multiple contributing factors and various symptoms. The highly significant overall positive association between galectin-3 and incident HF lends credence to its use as a tool to predict and prevent HF onset. However, given a top-quartile-to-bottom hazard ratio of 1.32, it is unlikely that measuring levels of galectin-3 alone will provide a comprehensive predictive model. It has been demonstrated that, although exposure to galectin-3 is positively associated with the outcome of HF, adjusting for other biomarkers, such as B-type natriuretic peptide (used in diagnosing HF), reduces this strength association [26]. It is, therefore, plausible that a combination of biomarkers, if correctly identified, might comprise a robust predictive model of incident HF in populations without symptomatic CVD. The results of this meta-analysis make a case for the possible inclusion of galectin-3 in that model. Given galectin-3’s well-established role as an inducer of cardiac fibrosis, ventricular remodelling, and inflammation in animal models, this meta-analysis constitutes a worthwhile synthesis of research thus far conducted using human data. Further studies evaluating the interaction between several of these biomarkers in predicting incident HF are recommended. As this study did not evaluate measures of risk prediction (such as a c-index), direct extrapolation of this study’s results to clinical risk prediction is limited without further research.

The significant drop in between-study heterogeneity upon exclusion of the study with pre-existing CVD may be due to possible reverse causation. However, as there was only one study with data on this specific association in CVD patients, further research is recommended. Future studies would be encouraged to draw from populations without pre-existing cardiovascular disease and to include raw data for adjustment standardization.

## CONCLUSION

There is a significant positive association between top-quartile log-galectin-3 levels and the risk of incident heart failure in community-based populations—however, notable heterogeneity between studies due to limited relevant published research warrants additional evidence. In particular, further studies on the association between galectin-3 and incident HF in studies without pre-existing CVD, further studies adjusting for other biomarkers implicated in HF risk, and further studies evaluating measures of risk association would help optimize the utility of galectin-3 as a predictive tool in clinical settings. This study herein demonstrates a highly conserved, statistically significant relationship between galectin-3 and incident heart failure. The next step is to investigate whether galectin-3 is a causal agent in associated pathophysiological processes, a necessary foundation for the development of possible galectin-3-based heart failure therapeutics and the early prediction and prevention of cardiac dysfunction.

## Figures and Tables

**Appendix I:** Newcastle-Ottawa quality assessment scale for cohort studies34.

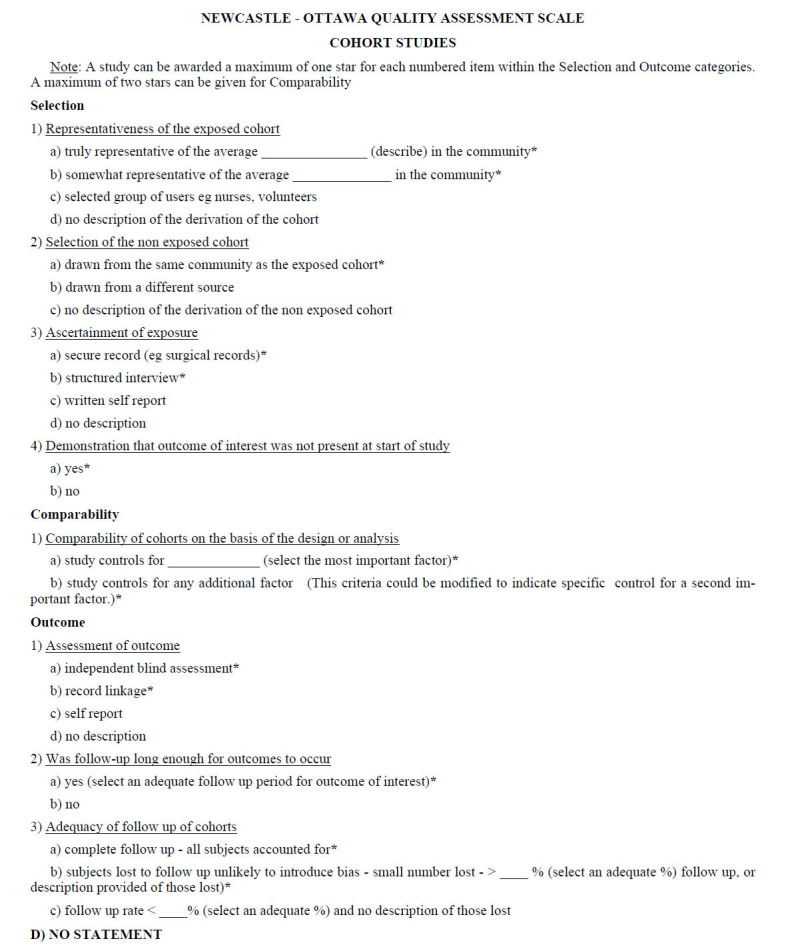

**Appendix II:** Newcastle-Ottawa Scale (NOS) quality assessment of included case-control and cohort studies.

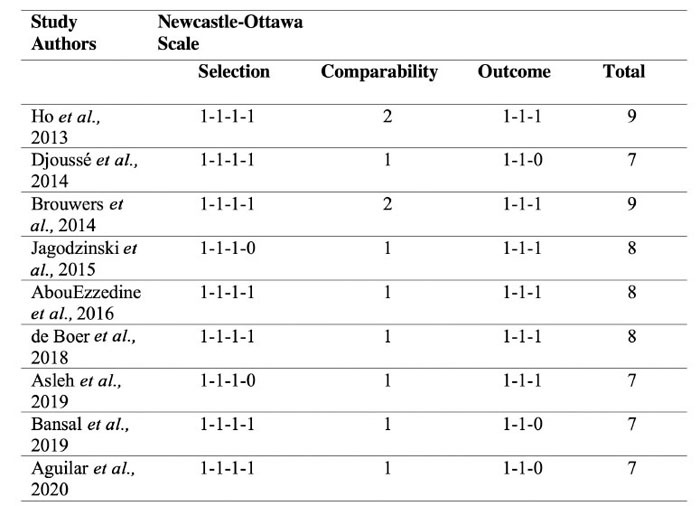

**Appendix III:** Hazard ratios converted to 4th-to-bottom quartile of log-gal-3 and incident HF. Values not needing conversion are presented in their original form.

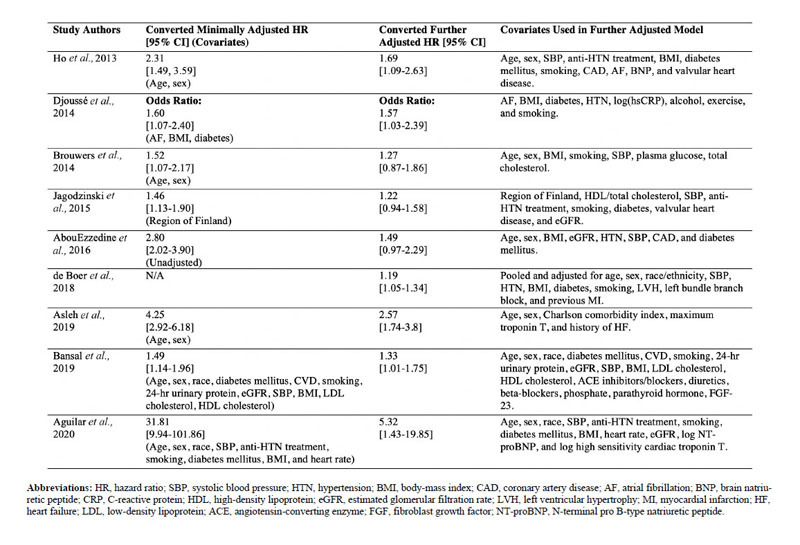

**Fig. (4):**
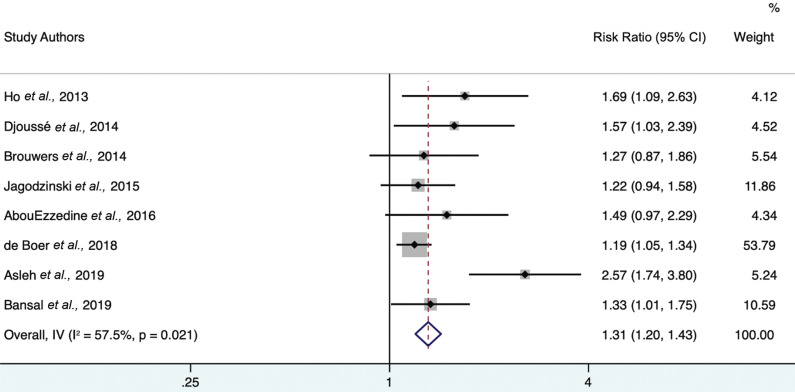
Forest plot of further adjusted model hazard ratios for the association between 4th-quartile-to-bottom-quartile log-gal-3 and incident HF, excluding the outlier Aguilar *et al*., 2020. Study weights (represented by the grey boxes) are from the random-effects analysis. 95% confidence interval for the heterogeneity statistic I^2^: (0-78.8).

**Table 1:** Study inclusion criteria.

**Inclusion Criteria**
Consists of primary research Includes the exposure galectin-3 Includes the outcome incident HF Reports a measure of association (OR, HR, RR) HF clearly defined and diagnosed Community-based population Conducted on human subjects English language Full-text freely available to the University of Cambridge

**Table 2:** Characteristics of included studies.

**Study Authors**	**Study ** **Design**	**Study Period**	**Geographical ** **Location(s)**	**Population Source(s)**	**Sample Size**	**Mean Age ± SD**	**% Female**	**% Diabetics**
Ho *et al*., 2013 [21]	Prospective Cohort	1996 - 2008	USA	Community-based (FHS)	3353	59	53	9.5
Djoussé *et al*., 2014 [22]	Prospective Nested Case-Control	1997 - 2011	USA	Community-based (PHS)	924	58.3 ± 8.1	0	5.2
Brouwers *et al*., 2014 [23]	Prospective Cohort	1998 - 2010	The Netherlands	Community-based (PREVEND-based on CVD risk)	8569	49	50	1.4
Jagodzinski *et al*., 2015 [24]	Prospective Cohort	1997 - 2012	Finland	Community-based (FINRISK)	8312	48.8 ± 22.1	49.2	5.9
AbouEzzedine *et al*., 2016 [25]	Prospective Cohort	1997 - 2000	USA	Community-based	1614	N/A	N/A	N/A
de Boer *et al*., 2018 [15]	Prospective Cohort	1989 - 2002	USA and the Netherlands	Community-based (FHS, CHS, PREVEND, and MESA)	22,756	60 ± 13	53.1	20
Asleh *et al*., 2019 [18]	Prospective Cohort	2002 - 2012	USA	Community-based CVD patients	1342	67.1	38.7	23.6
Bansal *et al*., 2019 [19]	Prospective Cohort	2003 - 2014	USA	Community-based (CRIC)	3314	57.5 ± 11.1	46	47
Aguilar *et al*., 2020 [26]	Prospective Cohort	1996 - 2013	USA	Community-based (ARIC)	4829	74.8	62.4	29.3

**Abbreviations:** FHS, Framingham Heart Study; PHS, Physicians’ Health Study; PREVEND, Prevention of Renal and Vascular End-Stage Disease; FINRISK, a large Finnish population survey; CHS, Cardiovascular Health Study; MESA, Multi-Ethnic Study of Atherosclerosis; CVD, Cardiovascular Disease; CRIC, Chronic Renal Insufficiency Cohort; ARIC, Atherosclerosis Risk In Communities.

**Table 3:** Characteristics of study outcomes.

**Study Authors**	**Defining Outcome of Interest ** **(Incident HF)**	**Number of Events**	**Follow-Up Length in Years**	**Outcome Collection**	Outcome Ascertainment*
Ho *et al*., 2013	HF, as confirmed by a panel of 3 physicians systematically reviewing records	166	8.1 (Mean)	Outpatient and hospitalization records	Framingham criteria
Djoussé *et al*., 2014	Self-reporting of HF by patients (who are themselves, physicians)	462	20+ (Maximum)	Annual follow-up questionnaires and (limited) chart review	Framingham criteria
Brouwers *et al*., 2014	HF, as identified by researchers using ESC guidelines	374	12.5 (Median)	Outpatient and hospitalization records	ESC guidelines
Jagodzinski *et al*., 2015	HF as identified by researchers using MORGAM/AHA criteria	641	15 (Maximum)	Finnish national health care registry	MORGAM and AHA criteria
AbouEzzedine *et al*., 2016	HF as defined by direct physician diagnosis or by trained nurse abstractors from medical records using Framingham criteria/ICD-9 codes	173	11 (Median)	Rochester Epidemiology Project medical records	Framingham criteria, ICD-9 code 402 or 428
de Boer *et al*., 2018	FHS: HF, as confirmed by a panel of 3 physicians systematically reviewing records CHS: Physician diagnosis of patients during follow-up META: HF as indicated by death certificates, hospitalization records, or interviews PREVEND: HF as identified by researchers using ESC guidelines	2095	12 (Mean)	FHS: Outpatient and hospitalization records CHS: Extensive physical and laboratory examinations and follow-up MESA: Death certificates, hospitalization records, interviews PREVEND: Outpatient and hospitalization records	FHS: Framingham criteria CHS: CHS criteria, ICD-9 codes 427-429.9 MESA: Identified from records PREVEND: ESC guidelines
Asleh *et al*., 2019	HF, as identified by researchers from records using Framingham criteria/ICD-9 codes	368	5.4 (Mean)	Rochester Epidemiology Project medical records	Framingham criteria, ICD-9 code 428
Bansal *et al*., 2019	2 researchers agreed upon a “probable” or “definite” HF diagnosis from follow-up questionnaires and medical records	477	7.9 (Median)	Hospitalization records and biannual questionnaire	“accepted standardized clinical criteria” (unspecified)	
Aguilar *et al*., 2020	HF hospitalizations adjudicated by experts, and trained personnel abstracted medical/death records for HF	1599	18 (Median)	Hospitalization records and annual participant reporting	ICD-9 code 428	

**Note:** *For Framingham criteria, see McKee *et al*., 1971 [31]. For ESC guidelines, see Ponikowski *et al*., 2016 [32]. For CHS criteria, see Fried *et al.*, 1991 [33]. The ICD-9 (International Classification of Diseases, Ninth Revision) is an internationally adopted classification scheme used to collect, process, classify, and present worldwide mortality statistics [27]. Diagnosis of HF derived from the MORGAM and AHA criteria, in conjunction with Finnish national health records, has been demonstrated to be of high specificity and validity [28].

**Abbreviations:** HF, Heart Failure; ESC, European Society of Cardiology; AHA, American Heart Association; ICD-9, International Classification of Diseases, Ninth Edition; FHS, Framingham Heart Study; CHS, Cardiovascular Health Study; MESA, Multi-Ethnic Study of Atherosclerosis; PREVEND, Prevention of Renal and Vascular End-Stage Disease.

**Table 4:** Characteristics of study assays.

**Study Authors**	**Sample Type**	**Storage Duration (Years)**	**Storage Temperature (°C)**	Galectin Measurement Method*	**Assay Manufacturer**
Ho *et al*., 2013	Plasma	14-16	-70	ELISA	BG Medicine, Waltham, MA
Djoussé *et al*., 2014	Plasma	20	N/A	ELISA	R&D Systems, Minneapolis, MN
Brouwers *et al*., 2014	Plasma	16	-80	ELISA	BG Medicine, Waltham, MA
Jagodzinski *et al*., 2015	N/A	20	-70	CMIA	Abbot Diagnostics, Abbott Park, IL
AbouEzzedine *et al*., 2016	N/A	N/A	N/A	ELISA	BG Medicine, Waltham, MA
de Boer *et al*., 2018	N/A	N/A	N/A	ELISA	BG Medicine, Waltham, MA
Asleh *et al*., 2019	Plasma	7-17	-70	ELISA	BG Medicine, Waltham, MA
Bansal *et al*., 2019	Plasma	6-11	-70	ELISA	R&D Systems, Minneapolis, MN
Aguilar *et al*., 2020	Plasma	4-20	-70	CMIA	Abbot Diagnostics, Abbott Park, IL

**Note:** *An enzyme-linked immunosorbent assay (ELISA) is a highly sensitive test that detects and quantifies substances, including antibodies, antigens, proteins, and hormones, in biological samples [29]. A chemiluminescent immunoassay (CMIA) is another type of immunoassay used for detecting substances in biological samples wherein the label (the indicator of the reaction) is a luminescent molecule [30].

**Table 5:** Main findings of included studies. *Converted values can be found in Appendix VI [31-34].

**Study Authors**	**Minimum Adjusted Model HR [95% CI] (Covariates)**	**Further Adjusted Model HR [95% CI]**	Comparison*	**Covariates in Further Adjusted Model**
Ho *et al*., 2013	1.39 [1.17-1.65] (Age, sex)	1.23 [1.04-1.47]	1 SD log-Gal-3 increase and incident HF	Age, sex, SBP, anti-HTN treatment, BMI, diabetes mellitus, smoking, CAD, AF, BNP, and valvular heart disease.
Djoussé *et al*., 2014	Odds Ratio: 1.60 [1.07-2.40] (AF, BMI, diabetes)	Odds Ratio: 1.57 [1.03-2.39]	4th quartile of log-gal-3 compared to the bottom quartile and incident HF	AF, BMI, diabetes, HTN, log(hsCRP), alcohol, exercise, and smoking.
Brouwers *et al*., 2014	1.18 [1.03-1.36] (Age, sex)	1.10 [0.95-1.28]	Linear gal-3 doubling and incident HF	Age, sex, BMI, smoking, SBP, plasma glucose, total cholesterol.
Jagodzinski *et al*., 2015	1.46 [1.13-1.90] (Region of Finland)	1.22 [0.94-1.58]	4th quartile of gal-3 compared to the bottom quartile and incident HF	Region of Finland, HDL/total cholesterol, SBP, anti-HTN treatment, smoking, diabetes, valvular heart disease, and eGFR.
AbouEzzedine *et al*., 2016	1.50 [1.32-1.71] (Unadjusted)	1.17 [0.99-1.39]	1 SD log-Gal-3 increase and incident HF	Age, sex, BMI, eGFR, HTN, SBP, CAD, and diabetes mellitus.
de Boer *et al*., 2018	N/A	1.07 [1.02-1.12]	1 SD log-Gal-3 increase and incident HF	Pooled and adjusted for age, sex, race/ethnicity, SBP, HTN, BMI, diabetes, smoking, LVH, left bundle branch block, and previous MI. (CHS: Adjusted for age, sex, race/ethnicity, previous MI, BMI, anti-HTN treatment, SBP, smoking, LVH, left bundle branch block, and diabetes).
Asleh *et al*., 2019	3.46 [2.51-4.78] (Age, sex)	2.25 [1.61-3.15]	3rd tertile of gal-3 compared to bottom tertile and incident HF	Age, sex, Charlson comorbidity index, maximum troponin T, and history of HF.
Bansal *et al*., 2019	1.17 [1.05-1.30] (Age, sex, race, diabetes mellitus, CVD, smoking, 24-hr urinary protein, eGFR, SBP, BMI, LDL cholesterol, HDL cholesterol)	1.12 [1.00-1.24]	1 SD log-Gal-3 increase and incident HF	Age, sex, race, diabetes mellitus, CVD, smoking, 24-hr urinary protein, eGFR, SBP, BMI, LDL cholesterol, HDL cholesterol, ACE inhibitors/blockers, diuretics, beta-blockers, phosphate, parathyroid hormone, FGF-23.
Aguilar *et al*., 2020	3.90 [2.47-6.17] (Age, sex, race, SBP, anti-HTN treatment, smoking, diabetes mellitus, BMI, and heart rate)	1.93 [1.15-3.24]	1 SD log-Gal-3 increase and incident HF	Age, sex, race, SBP, anti-HTN treatment, smoking, diabetes mellitus, BMI, heart rate, eGFR, log NT-proBNP and log high sensitivity cardiac troponin T.

**Abbreviations:** HR, hazard ratio; SD, standard deviation; HF, heart failure; SBP, systolic blood pressure; HTN, hypertension; BMI, body mass index; CAD; coronary artery disease; AF, atrial fibrillation; BNP, brain natriuretic peptide; CRP, C-reactive protein; HDL, high-density lipoprotein; eGFR, estimated glomerular filtration rate; LVH, left ventricular hypertrophy; MI, myocardial infarction; LDL, low-density lipoprotein; ACE, angiotensin-converting enzyme; FGF, fibroblast growth factor; NT-proBNP, N-terminal pro-B-type natriuretic peptide.

**Table 6:** Limitations of included studies.

**Study Authors**	**Study Limitations**
Ho *et al*., 2013	Relatively few HF events, predominantly white study population and Gal-3 sample storage length may degrade samples.
Djoussé *et al*., 2014	Lack of serial gal-3 measurement (only baseline), relatively small sample size, case-control design cannot eliminate survival bias, white-male-only subjects limit the generalizability of findings, Gal-3 sample storage length may degrade samples, outcomes ascertained *via* self-reporting (even if validated), lack of a standardized minimally adjusted model for cross-study analysis (age/sex or the like), no quantifiable mention of subject loss during follow-up.
Brouwers *et al*., 2014	Predominantly white study population, no serial gal-3 measurement (only baseline), and Gal-3 sample storage length may degrade samples.
Jagodzinski *et al*., 2015	Lack of serial gal-3 measurement (only baseline), samples stored for decades susceptible to degradation, p-value just above significance threshold, lack of a standardized minimally adjusted model for cross-study analysis (age/sex or the like), no explicit mention of incident HF present in the population at the start of the study.
AbouEzzedine *et al*., 2016	Lack of descriptive statistics for HF incidence cohort (subgroup within the overall study), lack of assay storage information, predominantly white study population, relatively smaller sample size compared to other community cohorts, p-value just above significance threshold, Gal-3 sample storage length may degrade samples, lack of a standardized minimally adjusted model for cross-study analysis (age/sex or the like).
de Boer *et al*., 2018	The duration between study enrollment and initial HF variable lacked serial galectin-3 measurement; pooled data comes from studies with different locations/researchers/variables that may confound results, lack of complete by-study analytics, lack of biomarker sample preservation information, lack of minimally adjusted models, Gal-3 sample storage length may degrade samples, MESA outcome ascertainment dependent upon conclusions of various medical records and not upon one uniform criterion or set of criteria.
Asleh *et al*., 2019	Predominantly white study population, effects due to residual confounding possible, Gal-3 sample storage length may degrade samples, no demonstration that the outcome of interest wasn’t present at the start of the study (analysis adjusted for prior HF, but patients were not excluded before analysis).
Bansal *et al*., 2019	Baseline exclusion by HF determined by self-reporting, incident HF based only on HF hospitalization, Gal-3 sample storage length may degrade samples, lack of a standardized minimally adjusted model for cross-study analysis (age/sex or the like), no quantifiable mention of subject loss during follow-up, criteria for outcome ascertainment unspecified.
Aguilar *et al*., 2020	Gal-3 samples stored for 15+ years are susceptible to degradation, bias due to patient loss in follow-up, lack of a standardized minimally adjusted model for cross-study analysis (age/sex or the like), and no quantifiable mention of subject loss during follow-up.

## LIST OF ABBREVIATIONS

HF: Heart Failure
CVD: Cardiovascular Disease
SWIFT: Sciome Workbench for Interactive Computer-Facilitated Text-mining
ELISA: Enzyme-Linked Immunosorbent Assay
CMIA: Chemiluminescent Immunoassay

## SUPPLEMENTARY MATERIAL

PRISMA checklist is available on the publisher’s website along with the published article.
